# Resorcylidene Aminoguanidine (RAG) Improves Cardiac Mitochondrial Bioenergetics Impaired by Hyperglycaemia in a Model of Experimental Diabetes

**DOI:** 10.3390/ijms12118013

**Published:** 2011-11-16

**Authors:** Magdalena Labieniec-Watala, Karolina Siewiera, Zofia Jozwiak

**Affiliations:** Department of Thermobiology, University of Lodz, 141/143 Pomorska Street, 90-236 Lodz, Poland; E-Mails: ksiewiera@gmail.com (K.S.); zjozwiak@biol.uni.lodz.pl (Z.J.)

**Keywords:** RAG (β-resorcylidene aminoguanidine), mitochondrial respiratory, streptozotocin diabetes, oxygraph Oroboros

## Abstract

Diabetes is associated with a mitochondrial dysfunction. Hyperglycaemia is also clearly recognized as the primary culprit in the pathogenesis of cardiac complications. In response to glycation and oxidative stress, cardiac mitochondria undergo cumulative alterations, often leading to heart deterioration. There is a continuous search for innovative treatment strategies for protecting the heart mitochondria from the destructive impact of diabetes. Aminoguanidine derivatives have been successfully used in animal model studies on the treatment of experimental diabetes, as well as the diabetes-driven dysfunctions of peripheral tissues and cells. Considerable attention has been paid particularly to β-resorcylidene aminoguanidine (RAG), often shown as the efficient anti-glycation and anti-oxidant agent in both animal studies and *in vitro* experiments. The aim of the present study was to test the hypothesis that RAG improves oxidative phosphorylation and electron transport capacity in mitochondria impaired by hyperglycaemia. Diabetes mellitus was induced in Wistar rats by a single intraperitoneal injection of streptozotocin (70 mg/kg body weight). Heart mitochondria were isolated from healthy rats and rats with streptozotocin-diabetes. Mitochondrial respiratory capacity was measured by high resolution respirometry with the OROBOROS Oxygraph-2k according to experimental protocol including respiratory substrates and inhibitors. The results revealed that RAG protects the heart against diabetes-associated injury by improving the mitochondrial bioenergetics, thus suggesting a possible novel pharmacological strategy for cardioprotection.

## 1. Introduction

Recent studies have shown that one of the major consequences of diabetes mellitus is the development of diabetic cardiomyopathy. Cardiovascular disease is the leading cause of death in the diabetic population. Cardiac dysfunction is common in diabetics regardless of risk factors for coronary artery disease and hypertension [1]. Emerging evidence suggests that diabetic cardiomyopathy is linked to alterations in myocardial fuel and energy metabolism. Different experimental data indicate that the mitochondrion is one of the main sites implicated in the development of cardiac dysfunction. Because mitochondria are the main cellular source of ATP, attention has been paid to understanding the causes of the decrease in mitochondrial respiratory function and the effect of the resulting decrease in ATP production on cardiac contractility and hemodynamics. Disruption of the mitochondrial respiratory function is regarded as a key factor in the development of pathologic complications in heart and other tissues under diabetic conditions [2]. Therefore, mitochondrial damage has been proposed as a central mediator in the development of diabetes complications. A number of reports have provided evidence for alteration in the respiratory activity of mitochondria isolated from different diabetic tissues including liver, heart, skeletal muscle and kidney. However, the consensus is not univocal as discrepant and often contradictory results in mitochondrial functions of diabetic tissues have been reported by different groups. It has been suggested that the decrease in mitochondrial respiration observed in diabetic tissues is due to a decrease in the electron transport chain activity. This decrease has been attributed to defects in several active sites of the mitochondrial electron transport chain, including all mitochondrial complexes (I, II, III, IV, and the ATP synthase) [3].

To understand the physiological and pathological changes of this complex disease, animal models of diabetes are important research tools, since animal studies allow for tight control over experimental conditions, which is almost impossible to achieve in human populations.

In the present study, we used STZ-induced rats as type 1 diabetes mellitus model. STZ-induced diabetic rats, commonly used as an animal model of type 1 diabetes mellitus, are obtained after selective destruction of β-cell by streptozotocin (STZ), a broad spectrum antibiotic, with diabetogenic effects. STZ-injected rats present many characteristics seen in insulin-dependent diabetic human patients: hypoinsulinemia, hyperglycaemia, ketonuria, and hyperlipidaemia. Therefore, this model is of great use to evaluate the changes promoted by uncontrolled type 1 diabetes [4].

For our study, we focused on the heart and, in particular on cardiac mitochondria. Heart in rats with acute streptozotocin-induced diabetes is characterized by altered metabolism and alterations in respiration and phosphorylation rates. Consequently, even small changes observed in the mitochondrial functionality can disturb the production of ATP. The loss of this valuable fuel for the heart is dangerous and can be cause of serious heart complications. Therefore, there is a continuous search for innovative treatment strategies for protecting the heart mitochondria from destructive impact of diabetes. The potential application of some agents to reduce glycation phenomenon and to increase the protective defense system by targeting mitochondria is very important.

In our opinion the role/activity of such compound could be fulfilled by β-resorcylidene aminoguanidine (RAG). Thus, the aim of the present study was to test the hypothesis that RAG improves oxidative phosphorylation and electron transport capacity in mitochondria impaired by hyperglycaemia. Aminoguanidine and its derivatives have been shown to inhibit AGE formation due to their reaction with carbonyl products that are formed during the end phases of non-enzymatic protein glycation, both *in vitro* and *in vivo* [5,6]. Nevertheless, the mechanism of RAG action is not understood precisely. Some data evidence that administration of RAG to diabetic rats (Wistar rats, RAG used at the dose of 10 mg·kg^−1^ by stomach probe, once daily for 8 weeks) decreases the level of AGE products (advanced glycation end products), glucose and triacylglycerols level, and the level of glycated Hb_A1c_ which take part in the development of post-diabetic complications in subjects suffering from diabetes [7]. Some other studies revealed that RAG acts as anti-oxidant and possesses antimutagenic and bacteriostatic properties [8].

Based on the above considerations, we have decided to check the impact of RAG on heart mitochondrial respirometric states believing that RAG is able to protect the mitochondrial bioenergetics against harmful effect of hyperglycaemia. To test this assumption, we used a streptozotocin diabetic rat model to examine the mitochondrial oxygen consumption as an index of mitochondrial respiratory function over a time course of 12 weeks of diabetes. The following specific aims were realized: (1) the determination of the level of ADP-coupled state respiration (STATE 3 or STATE P) as an index of the oxygen amount utilized on ATP production measured at saturating concentrations of ADP and P_i_. In other words it is an assessment of the capacity of the coupled oxidative phosphorylation (OXPHOS capacity) at the physiological ATP concentration; (2) the estimation of the level of STATE 4 respiration (state in the absence of ADP) as an index of mitochondrial condition/integrity after ATP production; (3) the evaluation of the FCCP-stimulated respiration (state E, uncoupled) as an index of the capacity of the respiratory electron transport system capacity (ETS); (4) the definition of the RCR (Respiratory Control Ratio) as an index of the mitochondrial capacity oxygen flux in the presence of ADP; (5) the designation of the LEAK Control Ratio as an index of mitochondrial uncoupling or mitochondrial dyscoupling at constant ETS capacity; (6) the valuation of the Phosphorylation System Control Ratio as an index of the limitation of OXPHOS capacity (State P) by the phosphorylation system; and (7) the assessment of the ADP/O flux ratio (nmol ADP/nAtm O) as an index of the amount of ADP utilization per 1 atom of oxygen [9]. The elucidation of these main points helped us to assess the general effect of RAG on rat heart mitochondria isolated from tested animal groups as described below.

## 2. Results and Discussion

**Figure 1:**
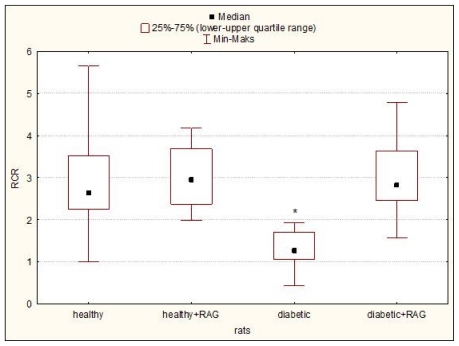
RCR (Respiratory Control Ratio) of rat heart mitochondria isolated from healthy and diabetic rats as well as from rats supplemented with RAG (40 mg/kg). Data presented as median and upper-lower quartile range, *n* = 12–17 animals. Significance of differences estimated by means of Kruskal–Wallis and median tests and *post hoc* all-pairwise comparisons Connover–Inman test: diabetic rats *vs*. all other tested animal groups: * *p* < 0.0001. For experimental details see “Experimental Methods”.

**Figure 2:**
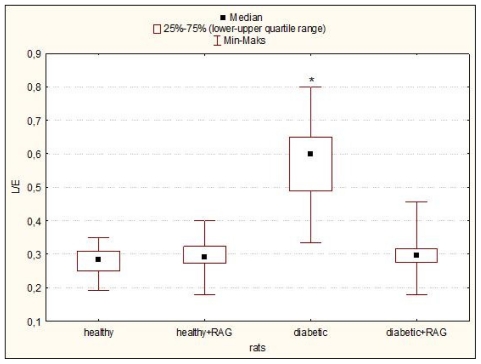
L/E (Leak Control Ratio) of rat heart mitochondria isolated from healthy and diabetic rats as well as from rats supplemented with RAG (40 mg/kg). Data presented as median and upper-lower quartile range, *n* = 12–17 animals. Significance of differences estimated by means of Kruskal–Wallis and median tests and *post hoc* all-pairwise comparisons Connover–Inman test: diabetic rats *vs*. all other tested animal groups: * *p* < 0.0001. For experimental details see “Experimental Methods”.

**Figure 3:**
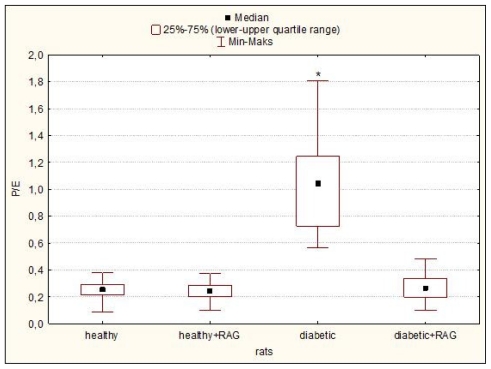
P/E (Phosphorylation System Control Ratio) of rat heart mitochondria isolated from healthy and diabetic rats as well as from rats supplemented with RAG (40 mg/kg). Data presented as median and upper-lower quartile range, *n* = 12–17 animals. Significance of differences estimated by one-way ANOVA and *post hoc* Tukey-Kramer test: diabetic rats *vs*. all other tested animal groups: * *p* < 0.001. For experimental details see “Experimental Methods”.

**Figure 4:**
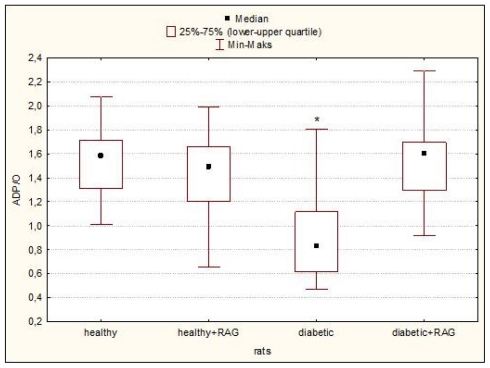
ADP/O ratio of rat heart mitochondria isolated from healthy and diabetic rats as well as from rats supplemented with RAG (40 mg/kg). Data presented as median and upper-lower quartile range, *n* = 12–17 animals. Significance of differences estimated by one-way ANOVA and *post hoc* Tukey-Kramer test: diabetic rats *vs*. all other tested animal groups: * *p* < 0.001. For experimental details see “Experimental Methods”.

The effect of STZ-diabetes (12 weeks) and subsequent treatment with RAG (40 mg/kg) on the blood glucose level and selected respiratory parameters in hearts isolated from the male Wistar rats are detailed in Tables 1 and 2 and Figures 1–4. It was shown that RAG supplementation effectively reduces the glycaemia level compared to glucose level measured in animals with non-treated diabetes. Nevertheless, this limitation in this parameter does not reach the glycaemic condition characteristic for healthy rats (Table 1).

It may be seen that the significant decrease in such tested mitochondrial respirometric states as: state 3, state 4, state E and in the parameters such as RCR and ADP/O and increase in L/E and P/E parameters were revealed. These results confirmed that long-fasting (3 months) and not-treated diabetes affects the heart mitochondria bioenergetics. The observed changes in the main parameters of mitochondrial respiration would consequently lead to the limitation in ATP production and probably to the increase in the ROS formation. However, the diabetes-induced mitochondria changes are well described [4,10,11], therefore the main aim of this paper was not the confirmation of the results well known for a long time but the using RAG as the anti-diabetic drug in the experimental diabetes treatment and checking whether RAG is able to reduce the negative effect of diabetes on mitochondrial bioenergetics. We have focused our attention on the most important mitochondrial parameters such as state 3, state 4, state E and other ones such as RCR ratio, L/E ratio, P/E ratio or ADP/O flux ratio. All these parameters were measured as described under “Experimental Methods in the Section 3.4 Mitochondrial Respiratory Measurements”. Our results clearly indicate that RAG used in a dose of 40 mg/kg and administrated for 3 months of treatment efficiently protects mitochondria from the adverse impact of diabetes. It has been reported that RAG rather does not allow for the development of the harmful effect of diabetes on mitochondria than restores the functioning of mitochondria to the condition typical for healthy animals (Table 2 and Figures 1–4). Heart mitochondria isolated from diabetic subjects showed significantly depressed states 3, 4 and state E relative to hearts of diabetic subjects supplemented with RAG. These data (Table 2) indicate that diabetic mitochondria respire in a slower manner and consequently other processes related to mitochondrial respiratory are inhibited.

As shown in Figure 1, RCR ratio, which gives the information about the state of mitochondrial coupling, is significantly reduced for diabetic mitochondria compared to those isolated from the other tested animal groups (healthy rats; healthy rats supplemented with RAG and diabetic rats supplemented with RAG).

Leak control ratio (L/E) was used to assess the state of mitochondrial uncoupling. As shown in Figure 2, L/E ratio significantly increased only for diabetic mitochondria as compared to other mitochondrial preparations (*p* < 0.0001). These results clearly indicate that diabetic mitochondria were partly uncoupled after the 3 months of diabetes. Nevertheless, the exposure to RAG successfully protected the heart mitochondria against STZ-diabetes.

Another parameter, Phosphorylation System Control ratio (P/E) let us observe the impact of the phosphorylation system on the OXPHOS capacity in functioning mitochondria. If the value of this ratio is about 1, this means that the mitochondrial respiration is fully inhibited. In the case of our study, only for diabetic mitochondria we have observed a significant increase in the value of this parameter (median = 1,045; *p* = 0.000137), (Figure 3).

The last measured parameter was ADP/O ratio which informs about the amount of ATP produced per total oxygen consumed by mitochondria. As shown in Figure 4, diabetic mitochondria produced less ATP than other tested mitochondria. For diabetic mitochondria isolated from animals supplemented with RAG, the value of ADP/O is similar to this one received for mitochondria isolated from healthy rats. It clearly indicates that diabetes has not affected the mitochondrial functionality in this group of tested animals.

To summarize, our results strongly suggest that diabetic mitochondria present some mitochondrial dysfunctions while diabetic mitochondria derived from RAG-treated animal group possess all tested parameters at the level characteristic for healthy rats. This *in vivo* experiment showed that RAG treatment can be effective in reduction of mitochondrial dysfunction observed in diabetic subjects.

However, at this stage is not clear if similar situation could occur in humans suffering from mitochondrial disturbances during the course of this disease. More, we even cannot be sure that after the next *in vivo* study (in the same experimental conditions) we have a chance to obtain the same (or at least similar) results. It was well documented in the paper of Mujkosova *et al*. that seasonal differences in the heart activity are observed. Their results revealed that values of mitochondrial heart Mg^2+^-ATPase activity in the winter/spring-period (W/S-P) exceeded significantly (*p* < 0.05–0.001) those in the summer/autumn-period (S/A-P). A similar trend was also observed in hearts of animals with acute (8 days) streptozotocin diabetes. Such results indicate that seasonal differences may play a decisive role in the evaluation of properties and function of rat heart mitochondria [12]. It is also worth noting that seasonal changes can modify the effect of the tested compound. Therefore our promising results describe in this paper require further research and further confirmations.

Our data are not the first which indicate that RAG could be successfully used in the anti-diabetic treatment. Previously, the studies of Waczulikova *et al*. revealed that RAG possesses inhibitory activity in the process of the non-enzymatic glycation and oxidation of proteins [13]. RAG induced *in vivo* a significant diminution of glycated proteins and an increase in the fluidity of cardiac sarcolemma, consequently, it resulted in the normalization of an increased malondialdehyde level in kidney tissue in rats with streptozotocin-induced diabetes [14].

As for the RAG effect on functioning of mitochondria, we suggest that activity of RAG strictly depends on the used concentration/dose. So far, only one paper [15] describes the mitochondrial bioenergetic upon exposure to RAG activity. In our previous research [15], we monitored the mitochondrial membrane potential, membrane fluidity and the respiration capacity in rat liver mitochondria isolated from Wistar rats in the *in vitro* system. RAG was used at three selected concentrations: 50, 100 and 200 μM. Then, our findings revealed that RAG had the ability to: fluidize a mitochondrial lipid bilayer, cause a depolarization of mitochondrial membrane and limit oxygen consumption by mitochondria. Surprisingly, these data have revealed that, regardless of the used concentration, RAG does not influence the respiratory chain of mitochondria under the conditions of state 4. Thus, we were unable to parallel the depolarization and fluidizing activity of RAG by alterations in mitochondrial respiratory function. Nevertheless, in samples with ADP-supported respiration (state 3), we observed the slight but significant limitation of mitochondrial respiration at higher RAG concentrations of 100–200 μM. These results, on one hand, showed that the changes in the structure of mitochondrial membrane are evidently not sufficient to significantly impair the transport of electrons through complexes of respiratory chain. On the other side, we argued that RAG could contribute to the reduced transformation of ADP to ATP, as evidenced in that paper. These *in vitro* studies have led to the conclusion that RAG may affect mitochondrial function, and thus, it may have an impact on the overall image of impaired physiology under some experimental pathological conditions.

In this paper, we demonstrate that RAG can be successfully used in the animal model of experimental diabetes. We have not only confirmed the beneficial use of RAG in diabetes-induced pathology but we have also proved that, upon RAG treatment, heart mitochondria can be protected from the injurious effect of glucose excess in organism. As it was shown, no significant differences in the mitochondrial parameters were found between healthy rats and diabetic ones supplemented with RAG. This suggests that RAG seems to be a drug that can eliminate the negative effect of hyperglycaemia on heart mitochondria function if used in a proper dosage. In 2002, Liptakova *et al*., used RAG (10 mg/kg, 8 weeks) in order to study its impact on antioxidant level in plasma and the liver, lipid oxidation and selected biochemical parameters (glucose level, glycated Hb, fructosamine, cholesterol, *etc*.) in Wistar healthy rats as well as in rats with STZ-induced diabetes [7]. The received data showed that RAG administration has not improved the antioxidant status in diabetic subjects and had a moderate effect on the reduction of such glycation markers as glycated hemoglobin or fructosoamine. From our point of view, it is highly probable that RAG was applied at the too low dose in order to reveal its anti-glycation (or its anti-oxidant) activity. Moreover, our hypothesis concerning the appropriate usage of dose in the *in vivo* studies is partly evidenced by the study by Watala *et al*. The Authors applied RAG at the dose of 4 mg/kg in the model of experimental diabetes (Wistar rats) and found that, as a result of RAG administration (60 days), neither “early” protein glycation products nor advanced glycation end-products were considerably affected. Farther, at the used dose, chronic RAG treatment did not significantly inhibit plasma protein glycation in STZ-diabetic rats and blood platelet fructosamine. Furthermore, although they demonstrated a tendency that RAG reduced AGEs in blood plasma proteins, this reduction remained beyond statistical significance. In addition to these results, the Authors have found that RAG possesses the antithrombotic activity and therefore can be useful to treat diabetic complications [16].

However, the selection of an appropriate dose useful for pre-clinical as well as clinical studies is not an easy challenge. Speaking strictly in terms of significance, *in vivo* implications are not so clear because the effect of size cannot be quantitatively specified at the level of single cells, *i.e.*, in *in vitro* investigation. Therefore, the choice of the “well-working” dose for *in vivo* studies can be tested for a long time. Based on our findings, we suppose that RAG should be used at a dose higher than 10 mg/kg if we want to observe its anti-diabetic activity.

## 3. Experimental Methods

### 3.1. Chemicals

RAG (β-resorcylidene aminoguanidine.HCl; C_8_H_10_O_2_N_4_·HCl) has been synthesized as described elsewhere [15]. Briefly, the synthesis of RAG involved a simple condensation reaction. An equimolar amount of resorcylaldehyde was mixed with aminoguanidine hydrogen carbonate. The reaction was performed in ethanol/water (1:1, v/v ratio), and the reaction conditions were optimized with concentrated hydrochloric acid. After a short time, a crystalline precipitate of RAG chloride was formed. All other reagents and solvents used were of the highest analytical reagent grade and were purchased from Sigma (St. Louis, MO, USA).

### 3.2. RAG Supplementation—In Vivo Experimental Design

Seventy male rats of Wistar strain weighing approximately 250–300 g were used in this experiment. Before the commencement of the experiment, the animals were acclimatized to the laboratory conditions for a period of 2 weeks. They were maintained at an ambient temperature of 25 °C and 12/12 h of light/dark cycle. Animals were given standard commercial rat chow and water ad libitum and were housed under standard environmental conditions until treatment or sacrifice. The experiments were conducted in accordance with the Guide for the Care and Use of Laboratory Animals published by the US National Institute of Health (NIH Publication No. 85–23, revised 1985), as well as with the guidelines formulated by the European Community for the Use of Experimental Animals (L358-86/609/EEC) and the Guiding Principles in the Use of Animals in Toxicology (1989). All experiments were carried out under approval of an appropriate institutional local ethics committee.

The experimental animals were randomly divided into four groups: 2 groups—each of them including healthy animals (2 × 15 rats) and further 2 groups—each of them including animals with STZ-induced diabetes (2 × 20 rats).

Finally there were the following experimental groups:

1. healthy rats (non-diabetic) that received only fresh water;
2. RAG control group—healthy animals that were given RAG (ad libitum in water) in a dose of 40 mg/kg, once daily for 12 weeks;
3. diabetic rats—animals with streptozotocin-induced diabetes that received only fresh water;
4. treated group—animals with streptozotocin-induced diabetes that were given RAG (ad libitum in water) in a dose of 40 mg/kg, once daily for 12 weeks;

Diabetes was induced by an intraperitoneal injection of streptozotocin (STZ, dissolved in 0.1 mol/L citrate buffer, pH 4.5) in a dose of 70 mg/kg body weight. Diagnosis of diabetes was made on the basis of the non-fasting blood glucose concentration (measured in the morning hours, 08:00–10:00 AM). The animals with blood glucose concentrations higher than 16.7 mmol/L, were considered diabetic and included to the study. Each STZ-injected rat showing hyperglycaemia lower than 16.7 mmol/L at 72 h after injection was excluded from the study. The experiment on RAG effect (administered at a daily dose of 40 mg/kg) started after 7 days upon induction of laboratory confirmed diabetes. At the termination of the experiment the survivors in both groups were sacrificed and their blood and organs were collected for further biochemical analyses.

### 3.3. Blood Collection and Glucose Level Determination

In-life non-fasting blood glucose (measured always at 09:00–10:00 AM) was measured once in control non-diabetic animals and monitored weekly in all STZ diabetic animals under study. Blood was obtained from tail vessels by needle prick and tested using glucose strips or, when exceeding 600 mg%, with biochemical analyzer. The last determination of blood glucose was recorded at time preceding the critical event (within a week) and referred respectively to as terminal glucose.

### 3.4. Isolation of Rat Heart Mitochondria

Mitochondria were isolated according to the procedure of Ferko *et al*., with small modifications [10]. Briefly, hearts damped with small volume of ice-cold isolation solution (IS, containing in mmol/L: 250 mM sucrose, 0.5 mM EDTA, 10 Mm Tris and 1 g/L bovine serum albumin, pH = 7.4) were cut into small pieces with scissors and left together with 10 mL of IS containing the addition of dispase II (Sigma D 4693) 2.5 mg/g wet wt. (heart) on 10 min. Then, the pieces of heart were then transferred to a teflon/glas homogenizer and homogenized gently for 2–3 min. After centrifugation of homogenate at 800× *g* for 10 min (4 °C) the protease containing supernatant together with a part of mitochondria which were in a direct contact with the protease, was centrifuged at 4800× *g*, 10 min, 4 °C. Then, the pellet was resuspended in the same volume of IS but without protease, again homogenized (4800× *g*, 10 min, 4 °C) and spun down as previously described. The last centrifugation of pellet was done at the same conditions describe above. Finally, the pellet containing rat mitochondria was again resuspended in the ice-cold isolation solution (IS buffer, pH 7.4). Mitochondrial protein content was determined by the Bicinchoninic Acid Kit.

### 3.5. Mitochondrial Respiratory Measurements

Oxygen consumption of isolated mitochondria was determined polarographically at 37 °C with a Clark oxygen electrode, connected to a high resolution respirometry (OROBOROS, Oxygraph, Innsbruck, Austria) in a closed chamber with a magnetic stirring. The oxygen consumption rates were calculated as the time derivative trace (DatLab software for data acquisition and analysis, OROBOROS^®^). The respiration medium consisted of 110 mM sucrose, 60 mM K-lactobionate, 0.5 mM EGTA, 1 g/liter BSA essentially fatty acid free, 3 mM MgCl_2_, 20 mM taurine, 10 mM KH_2_PO_4_, 20 mM HEPES adjusted to pH 7.1 at 37 °C (buffer called MIR05) and catalase (280 IU/mL) (obtaining so called MIR06 buffer) was used [17]. The medium was equilibrated for 30 to 40 min with air in the oxygraph chambers and stirred at 540 to 560 rpm until a stable signal was obtained for calibration at air saturation.

The corresponding oxygen concentration was calculated from the digitally recorded barometric pressure and the oxygen solubility at 37 °C. The amplified signal was recorded on a computer with online display of the calibrated oxygen concentration and oxygen flux (negative time derivative of oxygen concentration). Instrumental background flux was measured in separate controls using the same medium without biological material.

*Respirometric regime*: isolated rat heart mitochondria (0.175 mg of protein/mL for each measurement of respiration) and respiratory substrate for complex II/respiratory inhibitor for complex I—succinate (10 mM)/rotenone (0.5 μM) were added to the standard respiration medium—MIR06 (2 mL). Further, the respirometric states were induced by addition of substrates for electron input into a specific respiratory complex (state 2: respiration remains slow, mainly compensating for the passive proton leak and inner membrane ion channels such as uncoupling proteins or the permeability transition pore), and finally titration of a low but saturating concentration of ADP (state 3: respiration is activated by the back-flow of protons into the matrix through the ATP synthase and the concomitant partial drop of the electrochemical proton gradient across the inner mitochondrial membrane). After exhaustion of ADP (0.25 mM) which is phosphorylated to ATP, respiration returns to a resting level (state 4: elevated above state 2 if ATPase activity recycles ATP to ADP). FCCP-uncoupled respiration was performed by adding 1.5–2 μM of FCCP (carbonyl cyanide *p*-trifluoromethoxyphenylhydrazone) to mitochondria energized with succinate. Finally, antimycin A at 2.5 μM was used in order to completely inhibit of complex III (building so called ROX state) (Residual Oxygen consumption remains) in electron transfer system (ETS). ROX is the respiration due to oxidative side reactions remaining after application of ETS inhibitors to mitochondrial preparation(s). The value of ROX was measured separately for every mitochondrial sample and then ROX was subtracted from the other designated states (state P, state T, state E).

### 3.6. Mitochondrial Parameters

Directly, the following mitochondrial respirometry parameters were measured using oxygraph:

- Leak respiration (State 2)—mitochondrial respiration in the presence of egzogenous added substrates and inhibitors,
- State 3 respiration (or State P)—it is the ADP stimulated respiration of isolated coupled rat heart mitochondria in the presence of saturating ADP and Pi concentrations, supported by succinate as a substrate at saturating oxygen level,
- State 4 respiration (or State T)—it is the respiratory state obtained in isolated rat heart mitochondria after State 3, when added ADP is phosphorylated completely to ATP driven by electron transfer from respiratory substrates to O_2_.
- State E (ETS capacity)—it is a respiratory Electron Transfer System capacity of rat heart mitochondria in the experimentally induced non-coupled (fully uncoupled) state, in the mitochondrial preparations with succinate as a substrate. This state is induced by FCCP addition,
- ROX state—induced by Antymicin A addition.

Respiratory Control Ratio (RCR); L/E (Leak Control Ratio), P/E (Phosphorylation System Control Ratio) and ADP/O flux ratio were calculated according to the procedure described in [9,17].

- RCR (Respiratory Control Ratio)—is measured as State3/State 4,
- L/E ratio (Leak Control Ratio)—is measured as a ratio of “leak” respiration (State 2) to ETS capacity (State E),
- P/E (Phosphorylation System Control Ratio)—is measured as a ratio of State 3 (State P) to ETS capacity (state E),
- ADP/O (nmol ADP/nAtm O)—it informs how much mol of ATP is produced per mol of oxygen. It is calculated by measuring the decrease in oxygen concentration during the rapid burst of state 3 (State P) respiration after adding a known amount of ADP. It is necessary to subtract the basal respiration (State 2) due to imperfect coupling and the recycling of ATP. The change in concentration must then be multiplied by the chamber volume (2 mL). The quantity of oxygen in the chamber is calculated from published oxygen solubility data at the appropriate temperature.

### 3.7. Statistical Analysis

All the values from the tested animal groups (4) were expressed as a median and lower-upper quartile range. Data normality was checked using the Shapiro–Wilk’s test. Homogeneity of variance was evaluated using the Levene’s test. The statistical significance between tested groups was estimated using one-way ANOVA and *post hoc* Tukey-Kramer test for multiple comparisons (more than two groups). For data that showed non-homogeneity of variance, the non-parametric Kruskal-Wallis test, median test and *post hoc* all pairwise Connover–Inman test were used.

## 4. Conclusions

In summary, our present findings are the first to provide clear evidence that RAG can be used as the heart mitochondrial protective agent in the chronic experimental diabetes. This study showed that RAG successfully reduces harmful diabetes impact on mitochondrial respiratory maintaining its activity characteristic for mitochondria isolated from healthy (non-diabetic) rats. Although the mechanisms of the action of RAG on mitochondria may still remain elusive to some extent, our present data revealed that this agent can be used successfully under some pathological conditions where hyperglycaemia plays a significant role.

## Figures and Tables

**Table 1:** The impact of β-resorcylidene aminoguanidine (RAG) on glucose level in tested animal groups.

Parameter (mg%)	Healthy Rats	Healthy Rats +RAG	Diabetic Rats	Diabetic Rats +RAG	*p*
Glucose level in blood	114.5 (102; 111.75)	107 (103.5; 114.5)	536.5 (415.25; 674.25)	403.5 (362.25; 440.75)	* 0.024

Data were expressed as median and lower-upper quartile range;

* *p* = 0.024 for “diabetic rats” compared to “diabetic rats + RAG”.

**Table 2:** Changes in the heart mitochondria respiratory states in tested animal groups.

Parameter	Healthy Rats	Healthy Rats +RAG	Diabetic Rats	Diabetic Rats +RAG	*p*
State 3	838.25 (629.09; 1089.71)	914.15 (731.25; 1105.87)	400,72 * (351.77; 489.76)	873.91 (645.48; 1078.19)	* <0.001
State 4	309.21 (275.02; 343.41)	329.85 (276.83; 363.55)	179.91 * (168.18; 196.86)	287.88 (259.24; 327.95)	* <0.001
State E	1067.23 (908.23; 1244.42)	1086.07 (903.13; 1147.72)	529.31 * (414.27; 694.17)	968.05 (915.62; 1114.11)	* <0.001

Data were expressed as median and lower-upper quartile range;

* *p* < 0.001 for diabetic rats compared to the other animal groups; States: 3, 4 and E were measured as (pmol O_2_ × s^−1^ × cm^−3^); For experimental details see “Experimental Methods”.

## Abbreviations

AGEs: advanced glycation end products
DM: diabetes mellitus
ETS: electron transfer system
FCCP: carbonyl cyanide *p*-trifluoromethoxyphenylhydrazone
RAG: β-resorcylidene aminoguanidine
RCR: respiratory control ratio
STZ: streptozotocin
